# Perceptions of Tunisians on COVID-19 Vaccines: a qualitative study

**DOI:** 10.1192/j.eurpsy.2022.1339

**Published:** 2022-09-01

**Authors:** R. Jomli, H. Jemli, O. Sabrine, U. Ouali

**Affiliations:** 1 Razi Hospital, Psychiatry A, manouba, Tunisia; 2 university of tunis elmanar, Faculty Of Medicine Of Tunis, manouba, Tunisia; 3 El Razi, Avicenne, la mannouba, Tunisia

**Keywords:** perceptions, vaccin, Tunisia, Covid-19

## Abstract

**Introduction:**

In Tunisia, the Ministry of Health launched an awareness campaign in television spots and different social media platforms and started the vaccination campaign on the 13 March 2021 aiming to have vaccinated half of the Tunisian population by the end of 2021. However, to date, on July 31, 2021, only 1,104,286 people are completely vaccinated

**Objectives:**

The aim of the study was to identify Tunisians’ mental perceptions and attitudes towards COVID-19 vaccines to examine the predictors of the COVID-19 vaccine hesitancy in the Tunisian population.

**Methods:**

A group of citizens, randomly selected were invited to participate in the study. Data were collected through a focus group using a piloted topic guide. The entire discussion was recorded in audio-visual mode with a total duration of 1 hour. We also collected data on participant gender, age, education, and profession.

**Results:**

Seven women and four men participated in the study All participants reported having doubts about the efficacy of the vaccines. Two participants reported that their acquaintances died after being vaccinated. They suspected that expired vaccines have caused the reported deaths. *“I think these vaccines can be extremely dangerous. They could contain chemicals that are carcinogens or that have a castrating effect”*, an interlocutor stated, supported by the rest of the group. We found unanimously in our study, attesting to the relevance of religion in the lives of the Tunisian people, which is in agreement with literature

**Conclusions:**

Construction of multi-component and systematic interventions are required by public health authorities.

**Disclosure:**

No significant relationships.

